# Assessing Health Consequences of Vitamin D Fortification Utilizing a Societal Experiment Design: Methodological Lessons Learned from the D-Tect Project

**DOI:** 10.3390/ijerph18158136

**Published:** 2021-07-31

**Authors:** Mina Nicole Händel, Ramune Jacobsen, Fanney Thorsteinsdottir, Amélie Cléo Keller, Maria Stougaard, Camilla Bjørn Jensen, Caroline Moos, Katrine Sidenius Duus, Allan Jensen, Ulrik Schiøler Kesmodel, Bo Abrahamsen, Berit Lilienthal Heitmann

**Affiliations:** 1 Research Unit for Dietary Studies, The Parker Institute, Bispebjerg and Frederiksberg Hospital, 2000 Frederiksberg, Denmark; fanney.thorsteinsdottir@regionh.dk (F.T.); moos.caroline@gmail.com (C.M.); katrinesideniusduus@gmail.com (K.S.D.); berit.lilienthal.heitmann@regionh.dk (B.L.H.); 2 Department of Pharmacy, University of Copenhagen, 2100 Copenhagen, Denmark; ramune.jacobsen@sund.ku.dk; 3 Department of Public Health, University of Copenhagen, 1014 Copenhagen, Denmark; amelie.keller@sund.ku.dk; 4 Center for Early Intervention and Family Studies, Department of Psychology, University of Copenhagen, 1353 Copenhagen, Denmark; maria.stougaard@psy.ku.dk; 5 Center for Clinical Research and Prevention, Bispebjerg and Frederiksberg Hospital, 2000 Frederiksberg, Denmark; camilla.bjoern.jensen@regionh.dk; 6 Lifestyle, Reproduction and Cancer, Danish Cancer Society Research Center, 2100 Copenhagen, Denmark; allan@cancer.dk; 7 Department of Obstetrics and Gynaecology, Aalborg University Hospital and Aalborg University, 9000 Aalborg, Denmark; u.kesmodel@rn.dk; 8 Open Patient Explorative Network, Department of Clinical Research, University of Southern Denmark, 5000 Odense, Denmark; b.abrahamsen@physician.dk; 9 Department of Medicine, Holbæk Hospital, 4300 Holbæk, Denmark; 10 Nuffield Department of Orthopaedics, Rheumatology and Musculoskeletal Sciences, University of Oxford, Oxford OX3 7FY, UK; 11 The Boden Institute of Obesity, Nutrition, Exercise & Eating Disorders, University of Sydney, Sydney, NSW 2006, Australia

**Keywords:** vitamin D, pregnancy, chronic diseases, semi-ecological study

## Abstract

By utilizing historical changes in Danish legislation related to mandatory vitamin D fortification of margarine, which was implemented in the mid 1930s and abruptly abandoned in June 1985, the studies in the D-tect project investigated the effects of vitamin D on health outcomes in individuals, who during gestation were exposed or unexposed to extra vitamin D from fortified margarine. This paper reviews and narratively summarizes the analytic approaches alongside the results of the societal fortification experiment studies from the D-tect project and addresses the challenges in designing societal experiment studies and evaluating their results. The latter are discussed as lessons learned that may be useful for designers of similar studies, expected to be extensively utilized while researching the health consequences of the COVID-19 pandemic by comparing individuals born before and after the epidemic. In the D-tect project, 16 articles based on the societal fortification experiment were published analyzing 10 different outcomes and using different statistical approaches. Lessons learned included the detail of the analysis of the historical information on the exposure, availability and validity of the outcome data, variety of analytical approaches, and specifics concerning vitamin D effect evaluation, such as consideration of the influence of sunshine or season. In conclusion, the D-tect project clearly demonstrated the cost-effectiveness and research potential of natural- or societal-experiment-based studies.

## 1. Introduction

Public and environmental health researchers often use societal or natural experiment design studies to explore how unplanned natural or societal events (e.g., epidemics, famines, natural disasters, economic crises) or national policy changes affect health outcomes, or when alternative study designs are not possible or not ethical [1]. Among the most well-known examples of these studies are the Dutch famine studies that investigated the effects of extremely low macro- and micronutrient intake before or during gestation and early in life under famine on health outcomes later in life [2]. During World War II, a part of the Netherlands was cut off from food supplies for five months, resulting in rations below 1000 kcal/day/person. The Dutch famine studies later compared long-term health outcomes in individuals born in this part of the country before, during, or after the period of the famine. The studies utilized a life course approach to explore the role of early life factors on health throughout life. The Early Life Origins of Adult Disease hypothesis, also known as the Developmental Origin of Health and Disease (DOHaD) hypothesis, proposed in the 1930s [3], and later extended by Dubos [4], Widdowson and McCane [5], and Barker [6], was partially based on the results of the Dutch famine studies. Nowadays, attention is being drawn to the importance of prenatal nutrition and its influence on long-term health, examining the effects of specifics nutrients that may program poor health or diseases later in life. Among such nutrients are vitamins, in particular, the fat-soluble vitamin D.

In Denmark, from the mid 1930s and up until June 1985, it was required by law that all margarine products be fortified with vitamin D [7,8], after which it was abruptly banned. The changes in margarine fortification legislation constituted the basis of the societal experiment design studied in the D-tect project. Specifically, the risk of selected chronic diseases between cohorts of individuals born in Denmark before and after the abrupt change in fortification, and thus prenatally exposed or unexposed (depending on whether it was fortification initiation or cancelation) to extra vitamin D coming from fortified margarine, was compared [9]. While secular trends during 1983–1988 in the health outcomes examined, intake of margarine or the supplementation of vitamin D could have introduced residual confounding, a major strength of the D-tect study design was that factors such as maternal lifestyle, socioeconomy, and demography were in general equally distributed among individuals from both the exposed and the unexposed groups as all individuals were included from closely adjacent birth cohorts around the time of the policy change. Due to the design, where individuals from entire adjacent birth cohorts were examined, unmeasured covariates could be considered to be equally distributed between adjacent exposed and unexposed birth cohorts. Intake of margarine, based on food statistics for purchased margarine, was remarkably stable during the period, as were national policy recommendations for intake and supplementation of vitamin D.

This paper reviews the analytic approaches, results, and methodological challenges of the societal experiment design studies in the D-tect project. It discusses the lessons learned in designing the societal experiment studies to assess health programming effects generally and health programming effects related to vitamin D. The experience from the D-tect project may be useful for designers of similar studies, for instance, for research of the health consequences of the COVID-19 pandemic, by comparing individuals born before and after the epidemic.

## 2. Materials and Methods

The D-tect project comprised a battery of observational studies that used data from the Danish national registers to examine the health consequences of a historical change in the Danish margarine fortification legislation. We included all studies from the D-tect project that utilized the societal/natural experiment as their framework regardless of year of publication, study design, or outcome. In all the included studies, exposure was dichotomized into exposed and unexposed to the change in fortification practice, and seasonal variation in the vitamin D exposure were addressed in most.

The methods, results, and advantages and disadvantages of the methods used in the D-tect project’s fortification experiment studies published from 2013 are narratively presented. The studies are grouped by the investigated health outcomes and chronological order of their publication. Firstly, the studies analyzing the primary outcomes of type 1 diabetes, birth weight and childhood obesity, and childhood fractures are outlined, as these studies constituted the core of the D-tect project [9]. Afterward, the studies on secondary outcomes: gestational diabetes, pre-eclampsia, childhood asthma, and adult celiac and inflammatory bowel disease outcomes are presented, as the latter studies were designed and conducted after taking into consideration some of the experiences from the initial studies on the primary outcomes.

## 3. Results

In addition to the protocol paper [9], we have published 17 studies in the D-tect project on various outcomes. The primary outcomes were type 1 diabetes [10,11], birth weight and childhood obesity [12,13,14,15], and childhood fractures [16]. Secondary outcomes were gestational diabetes [17], pre-eclampsia [18,19], childhood asthma [20], adult celiac disease [21], inflammatory bowel disease [22], chance of live birth among women with fertility problems [23], and type 2 diabetes mellitus [24].

An overview of aims, participants, data sources, and statistical methods used in the included studies of the D-tect project can be found in Table 1 (where individuals were exposed/unexposed during gestation and early infancy) and Table 2 (where women were exposed/unexposed before or during pregnancy). 

### 3.1. Prenatal Vitamin D and the Risk of Type 1 Diabetes

Among the first published studies in the D-tect project were those examining the association between prenatal and first-year-of-life exposure to margarine fortification with vitamin D and the risk of type 1 diabetes (T1D) in children and adolescents [10]. The core date in the societal experiment design was 1 June 1985, when the legislation was issued to stop the mandatory fortification of margarine with vitamin D in Denmark [8]. The dataset was formed from an extract of the Civil Registration System [25], containing information on the date of birth and sex of more than 300,000 individuals born in Denmark during 1983–1988, linked to the Danish Registry of Childhood and Adolescence Diabetes [26], containing the date of the first diagnosis of verified T1D cases in Denmark (approximately 900 at any time during the study period). Previous studies have documented birth cohort effects for T1D risk (or secular trend) in individuals born in Denmark after 1985 [27]. Therefore, the approach of the societal experiment design study for T1D was linear spline regression of the secular trend, with regression cutoff points at the end of fortification and the end of the washout period (i.e., end of fortification plus 15 months); the month and year of birth determined exposure, and logged hazard ratio (HR) for T1D incidence was an outcome. Hypothesizing that changes in the regression line coefficients at the cutoff points would indicate the contribution or lack of fortification to the T1D risk, such changes were investigated, and no changes in T1D risk attributable to the fortification experiment were found. Identifying a relevant time frame for the washout period constituted a methodological challenge, as it was not known how long exactly fortified margarine stayed on the shelf or at people’s homes after the fortification policy changed; 6 months was considered appropriate. The methodological shortcoming of the approach was a short spectrum of investigated birth cohorts (6 years), and consequently, a relatively low number of T1D cases. A larger spectrum of birth cohorts would have allowed us to be more specific about the T1D secular trend changes over the years. Another methodological shortcoming, specific to vitamin D, was that, by this approach, seasonality of birth, essential when investigating environmental vitamin D effects, could not be considered. Another study was conducted to assess the effects of fortification on seasonality of birth in T1D cases, running stratified analyses among exposed (born 1983–1985) and unexposed (born 1986–1988) cohorts, and looking at odds ratios (ORs) for T1D incidence according to the predefined season of birth in males and females [11]. First, the ORs for T1D were found to be higher for individuals born in spring compared to that for those born in autumn in unexposed males, which supported the hypothesis, that extra vitamin D from fortified margarine minimized seasonal variation in vitamin D and, therefore, the risk of T1D in boys with late gestation during dark winter months [11]. Later, adjustment of regression models with meteorological data of monthly gestational sunshine hours (i.e., the sum of bright sunshine hours during the 9 months before the month of birth) eliminated seasonality of birth difference between exposed and unexposed cohorts of males [11]. Analytical simplicity approaching seasonality in the study (i.e., running several logistic regression models differentiated by exposure and sex groups) could be seen as a methodological shortcoming of this study.

### 3.2. Prenatal Vitamin D and Birth Weight and Childhood Obesity

Two studies [12,13] and two commentaries [12,14] concerning exposure to vitamin D–fortified foods and obesity risk in childhood were published. At the time of performing these studies, the available documentation (i.e., the Nordic Council of Ministers’ Report [28]) informed that, a mandatory vitamin D fortification of margarine in Denmark was started in 1961 and canceled in 1985, and a voluntary milk fortification with vitamin D in Denmark was started in 1972 and canceled in 1976 (0.25–0.38 μg vitamin D per 100 g milk). Based on this information, gestation cohorts were composed based on exposure and non-exposure to fortification (i.e., born during two years in or outside the fortification periods). The data on obesity were obtained from the Copenhagen School Health Records Register [29], comprising data from the school health examination of 372,636 children who attended schools in Copenhagen from 1936 to 2005. Linear regression modeling for birth weight and body mass index (BMI) at the age of 7, as well as logistic regression modeling for the risk of overweight and obesity at the age of 7, for exposed vs. unexposed, was performed in four analyses sets, i.e., taking into account margarine fortification initiation around 1961, milk fortification initiation around 1972, milk fortification cancelation around 1976, and margarine fortification cancelation around 1985 [12,13]. The analyses were not adjusted for any covariates except sex, based on the rationale that the individuals in the adjacent exposed and unexposed birth cohorts did not differ by anything but the exposure to vitamin D fortification status. The results of the multiple analyses were inspected for consistency, concluding that vitamin D fortification was not clinically relevant in relation to birth weight, nor did it affect body weight at 7 years [12,13]. Later, during the D-tect project, the history of margarine and milk fortification with vitamin in Denmark was investigated in detail, consulting multiple historical archived documents [7]. This investigation provided varying data on voluntary milk fortification, could not confirm the year of margarine fortification start (considered as January 1961), and specified the exact date of margarine fortification cancelation (1 June 1985). Even though the updated historical information did not essentially affect the interpretation of the already published results regarding gestational exposure to vitamin D fortification and the risk of childhood obesity, the scientific community was informed about the update of historical information [12,14]. The advantage of the analytical approach applied was the possibility to run multiple analyses around different time points of fortification change; the disadvantage was the misinformation concerning the history of fortification in the initial discovered historical document, the Nordic Council of Ministers’ Report [28].

Additionally, one study investigating seasonality of birth weight was published [15]. The study modeled birth weight according to the year and month of birth for the individuals from the Copenhagen School Health Records Register [29], born between 1936 and 1989, adjusting for gestational sunshine hours. Thus, this study, even though not addressing the question of fortification, could estimate whether seasonality of birth weight changed during the fortification period. In the case of attenuated seasonality during the fortification period, one could hypothesize that vitamin D from the fortified margarine prevented vitamin D deficiency during dark seasons, which influenced birth weight, and hence, fortification had an effect. Some seasonality patterns for birth weight were identified, which did not coincide with fortification changes. The advantage of the study was the use of the broad spectrum of birth cohorts, allowing the following of trends in birth weight for several decades, and a sophisticated seasonality modeling. The disadvantage of the study in relation to the vitamin D fortification effect analysis was that the modeling did not specifically address fortification.

### 3.3. Prenatal Vitamin D and the Risk of Fractures in Late Childhood

One study examined the effect of gestational exposure to vitamin D fortification on the risk of fractures among 10–18-year-old children born in Denmark during 1983–1988 [16]. The core date in the design was 1 June 1985, or cancelation of mandatory vitamin D fortification of margarine. Information on the incident and recurrent fractures was obtained from the Danish National Patient Register [30], and a multiplicative Poisson regression was used to examine the association between birth cohort and fracture rates in boys and girls, fitting a cohort–period–age model. The risk of fractures was found to be increased among both girls and boys from birth cohorts before 1985 (i.e., the vitamin D fortification termination): the risk ratio (RR) for exposed vs. unexposed girls was 1.15 (95 % CI 1.11, 1.20); for boys 1.11 (95 % CI 1.07, 1.14). When including the period effects in the model (i.e., exposed, washout, unexposed), the association was no longer significant. The potential modification effect (i.e., interaction) by the season of birth was explored and shown to be nonexistent. When stratifying the analyses by fracture site, the results did not change. Additionally, the risk of fractures by age in the chosen birth cohorts was explored. The strength of the cohort–period–age design employed in this study was a detailed exploration of the different aspects of the fracture risk (i.e., by sex, birth cohort, birth period, age, and season of birth), hence maximizing the utilization of the available data. Another important issue was the high validity of fracture diagnoses codes from the Danish National Patient Register, due to the low risk of error when diagnosing fractures by radiology in the hospital setting.

### 3.4. Prenatal Vitamin D and the Risk of Gestational Diabetes, Pre-Eclampsia, Childhood Asthma, Adult Celiac Disease, and Inflammatory Bowel Disease

**Figure 1:**
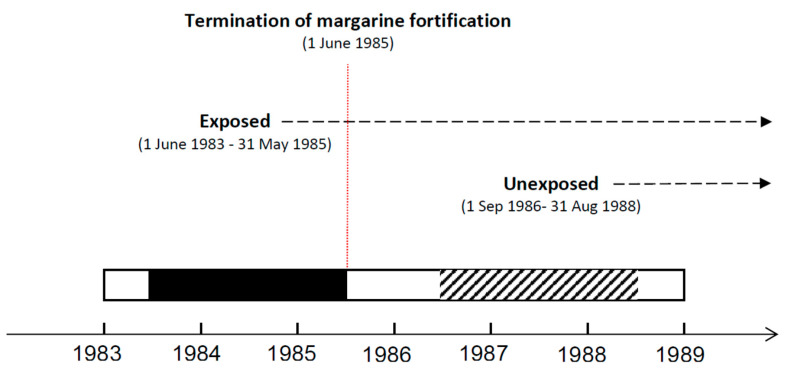
Design of the studies on fractures, gestational diabetes, pre-eclampsia, childhood asthma, adult celiac disease and inflammatory bowel disease (IBD) outcomes.

Studies analyzing the effect of gestational exposure to vitamin D fortified margarine on the risk of gestational diabetes [17], pre-eclampsia [18], childhood asthma [20], adult celiac disease [21], and inflammatory bowel disease (IBD) [22] later in life were subsequently published. The termination of the Danish fortification program in June 1985 constituted the basis of the study designs in these studies. The risk of the selected outcomes in birth cohorts from two years before (i.e., exposed) and after (i.e., unexposed) the fortification termination, separated by 15 months of washout period, was compared. Exposed and unexposed cohorts were followed for equal amounts of time in the Danish National Patient Register [30], to detect specified outcomes (see Figure 1).

For gestational diabetes and pre-eclampsia outcomes, the focus was on the first diagnosis of the relevant outcomes in women from the selected cohorts, who gave birth for the first time at age 14–27 years for pre-eclampsia [18] or at age 20–27 years for gestational diabetes [17]. These women were identified using information from the Danish Medical Birth Register [31], where information on covariates (e.g., mother’s smoking during pregnancy and pre-pregnancy BMI, offspring’s gestational age at birth and sex) was also obtained. For asthma outcomes, the focus was on the time of the first inpatient asthma diagnosis admission at age 0–9 years [20]. For celiac disease and IBD, the focus was on the first admission with a diagnosis at age 0–30 years [21,22. Due to the design, where individuals from entire adjacent birth cohorts were examined, unmeasured covariates were considered to be equally distributed between adjacent exposed and unexposed birth cohorts. Depending on the type of the outcome (i.e., time to the hospital-based first diagnosis, or the presence of the hospital-based diagnosis), Cox or logistic regression models were run, consequently running stratified analyses considered relevant (e.g., stratified by smoking status during pregnancy for pre-eclampsia [18] or by sex for asthma and IBD [20,22]). The effect of the season of birth was investigated by adjusting the analyses by month of birth (for asthma [20]), including exposure and season (or month) of birth interaction into regression modeling (for asthma [20], celiac disease [20], and IBD [22]), or running analyses stratified by season of birth (for gestational diabetes [17]). Prenatally exposed women born in spring, when pregnant later in life, had a lower risk of developing gestational diabetes compared to unexposed women born in the same season (OR 0.68, 95% CI 0.50, 0.94) [17]. Prenatally exposed women, if smoking when pregnant later in life, had a lower risk of developing pre-eclampsia compared to unexposed and smoking women (OR 0.49, 94% CI 0.34, 0.72) [18]. A decreased risk for asthma was shown in boys under 3 years of age exposed to the fortification, compared to boys of the same age not exposed to fortification (HR 0.78, 95% CI 0.67, 0.92) with no modification effect by month of birth [20]. ORs for developing celiac disease were similar in exposed and unexposed cohorts, with no significant modification effect by season of birth [21]. Lower odds of IBD were found for individuals under the age of 30 exposed to the fortification prenatally, compared to individual not exposed (OR 0.87, 95% CI 0.79, 0.95), and no significant effect of season of birth was detected [22]. The specific feature of these studies was the use of hospital-based diagnoses, thus leaving out mild and moderate asthma cases diagnosed in primary care [20] and raising some questions regarding the validity of the gestational diabetes diagnosis [17]. Another specific feature of all these studies (except the one on IBD, where the secular trend in disease incidence could partly explain the results [22]) was that no secular trends in the incidence of the investigated outcomes were identified in Denmark based on the obtained data from 1983 to 1988. It was not known, however, whether there were any secular changes over the longer period. Moreover, secular changes could occur in alternative exposure sources (i.e., vitamin D intake via food), and potential confounders (e.g., mother’s socioeconomic status, mother’s exposure to infections and physical activity), and the latter was explored if the data was available from the Danish Medical Birth Register [31]. The strengths of these studies were their novelty, long follow-ups, and large sample sizes with up to 200,000 individuals. 

### 3.5. Other Fortification Experiment-Based Studies in the D-Tect Project

Three studies were conducted exploring alternative hypotheses to “gestational exposure to vitamin D vs. health later in life” and utilizing the design of societal experiment with fortification. One study investigated the risk of pre-eclampsia in pregnant women exposed vs. unexposed to fortification; no fortification effect was shown [19]. Another study was on the probability of live birth in women with an infertility diagnosis registered in the Danish Infertility Cohort exposed vs. unexposed to fortification. The results showed that infertile women exposed to fortification had an increased chance of live birth compared to women not exposed to fortification [23].

Lastly, one study investigated the risk of type 2 diabetes in birth cohorts exposed to different doses of vitamin A from fortified margarine. The change in the dose of vitamin A margarine fortification in Denmark occurred early in 1961 [28]. The latter study showed an effect of an extra dose of vitamin A reducing the risk of type 2 diabetes later in life [24]. The methodological advantages and shortcomings of all the mentioned studies were similar to those described in the previous section.

## 4. Discussion

The described societal experiment studies present a semi-ecologic design, where the exposure is measured on an aggregated level (i.e., born during the period where margarine was or was not fortified), and outcome is measured at an individual level. A common advantage of semi-ecologic studies is that they are relatively inexpensive and can be conducted in a short time on large population samples. Moreover, it is a way to generate hypotheses, in particular if the hypotheses are relatively new. Finally, it is possible to examine the effect in whole or large populations, which is specifically relevant when examining rare outcomes or changes in national events/policies. The greatest limitation of semi-ecologic studies, similar to that of ecologic studies, is the risk of ecological fallacy, or making a wrong inference about associations at the individual level using observations of the group to which individuals belong [32]. If the intensity of the exposure is modest, as in the case of the studies based on margarine fortification experiment in the D-tect project (1.25 µg per 100 g of margarine supplying on average 13% (SD: 3–29%) of total dietary vitamin D [8]), and the exposure is not measured at the individual level, one can never fully exclude ecological fallacy. Thus, the associations identified in semi-ecologic design studies call for further investigation, ideally based on individual exposures, which constitutes the other battery of studies in the D-tect project. Furthermore, if there are secular trends or great variation in the outcome of interest, several decades of reporting is needed to help clarify the period effect. Nevertheless, cost-effectiveness and actuality of semi-ecologic, societal/natural experiment design studies in the presence of the COVID-19 epidemics will keep these types of studies on the research agenda, and the lessons learned during the D-tect project may be useful.

### 4.1. Historical Information

During the D-tect project, the first lesson learned while running the fortification-based design studies concerned the detail of the societal/natural experiment history. Initially, the studies were designed around the fortification experiment, the information on which was provided in a report from the Nordic Council of Ministers [28]. Even though the document seemed reliable, the detailed investigation of archived non-digitalized historical documents during the project [7] revealed that the fortification initiation date was not correct, which jeopardized the results of the studies on obesity outcomes already published. To avoid similar mistakes in forthcoming societal/natural experiment-based studies, an investigation of the detailed history concerning the event of interest from several sources (including both digital and physical archives) should be the first step. It is important to note that the job of investigating historical information in the D-tect project was not easy: both history and legislation documents concerning the events that happened 35–90 years ago, available both electronically and in the national library resources, were checked [7]. Despite the difficulty of enquiry, the detailed study of historical information when designing societal experiment-based studies is paramount.

Other societal events coinciding with the main event of interest and their effect on the outcomes should also be explored. In the D-tect study, other societal changes during the vitamin fortification period, affecting the quality of diet and vitamin D intake, could potentially have influenced the results. At the initiation of the D-tect project, societal changes other than margarine fortification, which could potentially affect the vitamin D status (e.g., national recommendations for supplementation of vitamins in the general Danish population), were not identified. However, subsequently, a change in the fiscal policy from 1 January 1987 to 31 December 1988 in Denmark, coinciding with dates included in the definition of the non-exposure group, was considered [33]. The fiscal policy was a tax reform aimed at reducing household expenditure and the ability to take up loans for both consumption and housing to increase the net savings of society. As a consequence of political reforms and financial crisis, the lifestyle and dietary quality could have changed, especially among vulnerable groups such as pregnant women [34]. The Chernobyl disaster also occurred around that time and the right and possibility for women to go on maternity leave in Denmark was modified in the 1980s, which might have been relevant and potentially affected pregnant women in Denmark. Thus, when running societal experiment design studies, one should study history and societal changes in detail and discuss the potential influences of both the event of interest and the contextual events. The assistance of historians and sociologists is highly recommended.

### 4.2. Methodological Issues on Data: Sources, Outcomes, and Covariates

The Dutch famine studies, exploring the consequences of poor nutrition very early in life for health later in life, would not be possible if the Netherlands had not kept high-quality health records for the entire nation. Similarly, the D-tect fortification design studies would not be possible without the administrative health registers in Denmark, covering all people legally residing in Denmark, where everyone can be identified via their unique personal identification number in registers with different information (demographic, socioeconomic, health etc.) which can be merged [35]. The Danish welfare and healthcare system’s national administrative and health registers were designed to fulfill administrative and economic purposes (e.g., to reimburse the hospitals). However, the national registers are extensively used for epidemiological research, maintaining ethical and data security requirements [36]. Thus, the feasibility of the societal/national experiment design studies depends on the country’s healthcare system and the availability of health data to researchers. Alternatively, with the widespread use of different health apps, one could design a study with random samples of exposed and unexposed persons and other than national health register data resources. In any case, the reliability and validity of the health outcome data is a fundamental issue, and it is always better to choose outcomes that are validated or do not raise serious validity questions. In the D-tect project, the majority of outcomes from the registers were either directly validated against medical records (e.g., birth weight, TD1, childhood asthma) or captured in areas where diagnosis was not challenging (e.g., hospital-treated fractures in the in- and outpatient setting).

Availability of data on covariates was also important in the D-tect project, even though the initial design idea was to compare all (i.e., unselected) individuals born in adjacent years, assuming that potential confounders/covariates would be equally distributed between the exposed and unexposed cohorts. The analysis of the T1D risk has shown secular trends in children born only up to 6 years apart, suggesting that the distribution of other characteristics and potential confounders could also change over time. Therefore, for pre-eclampsia and gestational diabetes studies, data on other covariates were obtained and included in the analyses. However, as expected, the results were essentially similar before and after adjusting for these variables. Thus, the lesson learned is that one needs to theoretically investigate the alternative risks as well as potential confounders in the associations investigated before obtaining the data, and consequently obtain all required data available.

### 4.3. Methodological Issues on Statistical Approaches

As already mentioned, the initial idea for the analyses in the D-tect fortification design studies was to compare the risk of selected chronic diseases in adjacent, as close as possible, exposed and unexposed birth cohorts, assuming that all other covariates or potential confounders would be equally distributed [9]. The discovery of the birth cohort effect documented for T1D cases for children born in Denmark around 1985 inspired us to look at different analytic approaches, and several of them were applied in subsequent studies: regression discontinuity [37] when analyzing T1D outcomes [10], age–period–cohort analyses [38] analyzing fracture outcomes [16], the approach similar to time series [39] analyzing birth weight seasonality [15]. For the latter outcome, the spectrum of the birth cohorts investigated was broad: slightly more than 50 years [15]. For T1D and fractures, this spectrum stretched only 6 years, thus making conclusions on regression changes or period effects less reliable [10,16]. Thus, the lesson learned is that when analyzing the effect of societal/natural events such as gestational exposures, it is always better to obtain a wide spectrum of birth cohorts, on both sides of the event of interest. The probability that the changes identified can be attributed to this societal or natural event rises with the period the analyses covers at the expense of being able to control for confounding.

Another lesson learned follows from the variety of regression modeling applied in the analyses presented: logistic, linear, spline linear, Cox, Poisson regression models; crude vs. adjusted, overall vs. stratified analyses. As semi-ecologic design studies face the ecologic fallacy risk [32], the hypotheses researched should stay robust in as many sensitivity tests as one can afford, and the more that are afforded, the better. Statistical competence in designing and running societal/natural experiment studies requires both analytical creativity to generate the ideas for statistical approach and computational ability to execute these ideas. Therefore, when running societal/natural experimental studies, the assistance of a statistician is highly recommended.

### 4.4. Lessons Concerning Studying Exposure to Vitamin D

Vitamin D is a somewhat unusual vitamin, because its main source is not only foods (e.g., oily fish, eggs), fortification, and supplementation but also synthesis in the body due to skin exposure to solar radiation from bright sunshine [40]. It can be argued that vitamin D is a vitamin only in winter and an endogenous compound in summer. In any case, to elucidate the effects of vitamin D fortification, one needs to consider exposure to bright sunshine. In the D-tect project, a couple of approaches were implemented to take gestational exposure to sunshine into account. The first was adjustment in the analytical models for environmental gestational sunshine hours (i.e., the sum of monthly bright sunshine hours during the 9 months before the month of birth, where information on monthly sunshine hours was obtained from the national meteorological institute). The second one was consideration of the season of birth in the regression modeling, e.g., by adjustment, interaction, or stratification. Both meteorological gestational sunshine hours and season of birth were of ecologic nature with related disadvantages. Nevertheless, consideration of ecologic information on sunshine in the D-tect fortification experiment studies was crucial (e.g., for the interpretation of fortification effects on the seasonality of birth in T1D cases). Compared to the relatively straightforward definition of gestational sunshine hours (i.e., obtaining information from the meteorological institute), the definition of the season of birth in the D-tect project underwent development. First, the seasons were defined a priori, where spring, summer, autumn, and winter were presented by three classic calendar months. Later, after obtaining and analyzing the distribution of meteorological sunshine hours, seasons were redefined by moving one month forward; however, sensitivity analyses with alternative season definitions were also run. Thus, the overall lesson learned would be to adjust vitamin D fortification analyses for sunshine in all possible ways according to available data. If the meteorological data are not available, the sensitivity of the results should be tested with different a priori definitions of the seasons.

## 5. Conclusions

Vitamin D fortification studies in the D-tect project demonstrate the cost effectiveness and research potential of societal/natural experiment studies, provided that historical information about the societal/natural events is valid and good quality data on health outcomes for large populations over longer periods of time are available. Sophisticated and multiple statistical approaches to examine specific research questions decrease the probability of committing the ecologic fallacy when interpreting results, a type of bias that ideally can only be eliminated by conducting further studies with an individualized exposure assessment.

## Figures and Tables

**Table 1:** Overview of aims, participants, data sources, and statistical methods used in the studies of the D-tect project, where individuals were exposed/unexposed during gestation and early infancy.

Outcomes	Aim	Participants	Data Source	Statistical Methods
Type 1 diabetes (T1D) [10,11]	To examine whether gestational and early infancy exposure to low-dose vitamin D from a mandatory margarine fortification program in Denmark influenced the risk of developing T1D before the age of 15 years.	Children aged 0–14 years	Civil registration numbers linked to the the Danish Childhood Diabetes Registry	Cox regression models
	To examine whether gestational and early infancy exposure to margarine fortification was associated with seasonality of birth in Danish T1D patients.	Children aged 0–14 years	Civil registration numbers linked to the the Danish Childhood Diabetes Registry. Meteorological data of monthly gestational sunshine hours.	Cox regression models
Birthweight and body size [12,13,14,15]	To study the impact of the Danish vitamin D fortification programs on birthweight.	Newborn	Copenhagen School Health Record Register	Linear and logistic regression models
	To investigate whether prenatal exposure to vitamin-D fortification of margarine and low-fat milk was also associated with body size at 7 years of age.	Children aged 7 years	Copenhagen School Health Record Register	Linear and logistic regression models
Fractures [16]	To examine the risk of fractures among adolescents from proximate birth cohorts born around the date of the termination of a mandatory national vitamin D fortification program.	Children aged 10–18 years	Civil registration numbers linked to the Danish National Patient Registry	Multiplicative Poisson models (age, period, cohort)
Gestational diabetes mellitus (GDM) [17]	To examine whether exposure during fetal life to extra vitamin D from food fortification was associated with a reduction in the risk of subsequently developing GDM and to examine whether the effect of the vitamin D from fortification differed by women’s season of birth.	Nulliparous women aged 20–28 years giving birth after gestational week 22 during the period from 2004 to 2016	Civil registration numbers linked to the Danish National Patient Registry and the Danish Medical Birth Register	Logistic regression models
Pre-eclampsia [18]	To examine whether fetal exposure to a small dosage of extra vitamin D from food fortification was associated with a decrease in the risk of pre-eclampsia later in life.	Nulliparous women aged 14–28 years giving birth after gestational week 22	Civil registration numbers linked to the Danish National Patient Registry	Logistic regression model
Asthma [20]	To examine whether children born to women exposed to the margarine fortification policy with a small dose of extra vitamin D during pregnancy had a reduced risk of developing asthma until age 9, compared to children born to unexposed women.	Children aged 0–9 years	Civil registration numbers linked to the Danish National Patient Registry	Cox regression models
Celiac disease (CD) [21]	To examine whether season of birth or prenatal exposure to extra vitamin D from food fortification were associated with developing CD later in life.	Individuals up to age 30	Civil registration numbers linked to the Danish National Patient Registry	Logistic regression models
Inflammatory bowel disease (IBD) [22]	To examine whether season of birth or prenatal exposure to extra vitamin D from food fortification were associated with developing IBD later in life.	Individuals up to age 30	Civil registration numbers linked to the Danish National Patient Registry	Logistic regression models
Type 2 diabetes mellitus (T2DM) [24]	To examine whether exposure during fetal life to extra vitamin A from food fortification was related to subsequent risk of developing T2DM in adulthood (before age 49).	Individuals aged between 36 to 49 years	Civil registration numbers linked to the Danish National Patient Registry and Danish National Diabetes Register	Logistic regression models

Abbreviations: Celiac disease (CD); Gestational diabetes melli-tus (GDM); Inflammatory bowel disease (IBD)Type 1 diabetes (T1D); Type 2 diabetes mellitus (T2DM).

**Table 2:** Overview of aims, participants, data sources, and statistical methods used in the studies of the D-tect project, with vitamin D exposure before or during pregnancy.

Outcomes	Aim	Participants	Data Source	Statistical Methods
Pre-eclampsia [19]	To examine whether exposure to extra vitamin D from food fortification was associated with a decrease in the risk of preeclampsia	All nulliparous women giving birth after gestational week 22 in the period 1 June 1983 to the 31 August 1988	Civil registration numbers linked to the Danish National Patient Registry	Logistic regression models
Live births in women diagnosed with infertility [23]	To examine the association between extra vitamin D from a mandatory margarine fortification program and chance of live birth among infertile women	All women diagnosed with primary infertility from 1 September 1986, to 31 August 1991. Any live birth for each woman in the cohort within a follow-up period of 12 months after the initial infertility diagnosis date.	Civil registration numbers linked to the Danish National Patient Registry, the Danish Medical Birth Register, the Danish Infertility Cohort, and the Danish In Vitro Fertilization Registry	Logistic regression models
